# Graph ODEs and Beyond: A Comprehensive Survey on Integrating Differential Equations with Graph Neural Networks

**DOI:** 10.1145/3711896.3736559

**Published:** 2025-08-03

**Authors:** Zewen Liu, Xiaoda Wang, Bohan Wang, Zijie Huang, Carl Yang, Wei Jin

**Affiliations:** Emory University, Atlanta, GA, USA; Emory University, Atlanta, GA, USA; Emory University, Atlanta, GA, USA; Amazon, Los Angeles, CA, USA; Emory University, Atlanta, GA, USA; Emory University, Atlanta, GA, USA

**Keywords:** Graph Neural Networks, Differential Equations, Deep Learning

## Abstract

Graph Neural Networks (GNNs) and differential equations (DEs) are two rapidly advancing areas of research that have shown remarkable synergy in recent years. GNNs have emerged as powerful tools for learning on graph-structured data, while differential equations provide a principled framework for modeling continuous dynamics across time and space. The intersection of these fields has led to innovative approaches that leverage the strengths of both, enabling applications in physics-informed learning, spatiotemporal modeling, and scientific computing. This survey aims to provide a comprehensive overview of the burgeoning research at the intersection of GNNs and DEs. We will categorize existing methods, discuss their underlying principles, and highlight their applications across domains such as molecular modeling, traffic prediction, and epidemic spreading. Furthermore, we identify open challenges and outline future research directions to advance this interdisciplinary field. A comprehensive paper list is provided at https://github.com/Emory-Melody/Awesome-Graph-NDEs.

## 1 Introduction

Understanding and predicting complex behaviors in natural and engineered systems is a fundamental challenge across scientific and industrial domains. Many real-world phenomena exhibit dynamic evolution over time, governed by intricate interdependencies between variables. Examples include climate patterns shaped by atmospheric and oceanic interactions [1], population dynamics influenced by birth and migration rates [2], financial markets driven by investor behavior and economic indicators [3], disease progression driven by biological factors [4], and the spread of infectious diseases determined by transmission dynamics and intervention strategies [5, 6]. Capturing these temporal changes and underlying mechanisms requires mathematical models that not only describe system behavior but also provide predictive insights.

To effectively model dynamical systems, Differential Equations (DEs), such as Ordinary Differential Equations (ODEs) [7], Partial Differential Equations (PDEs) [8], and Stochastic Differential Equations (SDEs) [9], relate one or more unknown functions to their derivatives, thus describing how outputs vary given changing variables. At their core, DEs consist of three essential components: (1) *state variables* that describe the system’s condition, (2) *derivatives* that model and capture the rate of change, and (3) *parameters* that influence the dynamics under given initial and boundary conditions. These elements work together to provide a structured approach to understanding how systems evolve over time.

Despite the crucial role of DEs in modeling complex phenomena, various challenges arise from real-world applications. Notably, many systems exhibit intricate, high-dimensional dynamics that are difficult to capture using purely knowledge-driven DE formulations [10], as deriving accurate governing equations often requires human expert involvement. Moreover, computational efficiency remains a major obstacle, especially for high-dimensional and non-linear PDEs, since traditional numerical solvers must manage an enormous number of equations corresponding to the system’s graph structure, often rendering these approaches prohibitively expensive [11–13]. In response to these challenges, neural differential equations (NDEs), such as Neural ODEs [14], have emerged as a data-driven alternative that learns the underlying dynamics directly from data, bypassing the need for explicit formulation of governing rules. This innovative approach enables the modeling of systems for which traditional equations may be intractable or unknown. Nevertheless, while NDEs excel at capturing temporal evolution, it remains challenging to model spatial dynamics, such as epidemic spread in social networks [15] or transportation flows in urban networks [16], where discrete interactions complicate continuous-state representations. This limitation urgently calls for methods that can effectively integrate temporal dynamics with spatial context.

To handle the above issues, recent research has leveraged Graph Neural Networks (GNNs) [17, 18], powerful tools for learning relational data, to build graph-based NDEs and model the complex interactions between variables. Early explorations integrate the graph learning capabilities of GNNs within the continuous-time framework of NDEs and propose Graph neural ODEs [19, 20], which offer a versatile and powerful approach to modeling complex systems that evolve over both space and time. This integration not only enables the capture of dynamic temporal behavior but also leverages the rich spatial relationships encoded in graph structures. Beyond Graph Neural ODEs, the broader class of Graph Neural Differential Equations (Graph NDEs), including Graph Neural PDEs [21] and Graph Neural SDEs [22], bridges the gap between NDEs and GNNs.

### Contributions.

In this work, we aim to present a comprehensive and latest review of methods that combine graph neural networks with differential equations, addressing the gap by summarizing key tasks, methodologies, and applications in this evolving field. Our contributions can be summarized as follows:

- We offer *the first comprehensive review* of Graph NDEs that model continuous spatial and temporal dynamics.
- We introduce a structured taxonomy of Graph NDEs in Section 3 and conduct an in-depth review of research integrating GNNs with different classes of differential equations, including ODEs, PDEs, and SDEs, as detailed in Section 4.
- We explore the diverse applications of Graph NDEs in Section 5, highlighting their impact across various real-world scenarios.
- We identify emerging trends, key challenges, and promising future research directions in Section 6, aiming to inspire further exploration in this interdisciplinary field.

### Connections to existing surveys.

While previous surveys have explored Graph NDEs, they often lack comprehensiveness in methodology and categorization, limiting their ability to fully bridge GNNs and NDEs. Many focus on specific applications of neural differential equations [23–25], overlooking spatial dynamics. Others examine the integration of GNNs with differential equations [26, 27] but remain narrow in scope regarding DE types and categorization. In contrast, our survey compiles a broad range of recent studies, offering a detailed review of methodologies, challenges, and applications. Additionally, we present a well-structured taxonomy as well as valuable insights for future research.

## 2 Background

### 2.1 Learning on Graphs

In this paper, we define a graph as 𝒢=(𝒱,ℰ), where |𝒱|=N represents the number of nodes, and ℰ⊆𝒱×𝒱 represents the set of edges connecting nodes. The features of all nodes is represented as $X=\left\{x_{1},x_{2},\ldots x_{N}\right\}\in R^{N\times D}$, where D denotes the feature dimension. The adjacency matrix of 𝒢 is denoted as A, where $A_{ij}=1$ if the edge $e_{ij}\in ℰ$ and $A_{ij}=0$ if $e_{ij}\notin ℰ$. GNNs provide a flexible framework to learn graph representations. A common paradigm is message passing, where each node v updates its representation $h_{v}$ based on aggregating messages from its neighbors 𝒩(v). A GNN with L layers can be described as:

(1) $$h_{v}^{\left(l+1\right)}=f_{\phi }\left(h_{v}^{\left(l\right)},\underset{u\in 𝒩(v)}{⨁}f_{\theta }\left(h_{v}^{\left(l\right)},h_{u}^{\left(l\right)},e_{uv}\right)\right),\forall l\in [L],$$

where $f_{\theta }$ and $f_{\phi }$ are learnable functions parameterized by θ and $\phi ,e_{uv}$ denotes edge features (if available), and ⨁ is permutation invariant aggregation operator that aggregates neighbor information. The final representation can then be used for downstream tasks such as link prediction and graph-level classification [28], etc.

### 2.2 Neural Differential Equations

Differential equations model dynamic systems across various domains, with their form varying based on the system. In the following, we illustrate three common types of DEs and NDEs.

#### Ordinary Differential Equations (ODEs).

ODEs describe system evolution with respect to a single independent variable, typically time t. The general form is: $\frac{dx}{dt}=f(x(t),t)$, where x(t) is the system state, and f dictates its rate of change.

#### Partial Differential Equations (PDEs).

PDEs involve multiple independent variables and their partial derivatives. A classical example is the diffusion equation, given by: $\frac{\partial u}{\partial t}=\alpha \nabla^{2}u$, where u=u(x,t) represents the unknown quantity varying in spatial coordinate x and time t,α is the diffusion coefficient, quantifying the rate of spatial dispersion, and $\nabla^{2}$ denotes the Laplacian operator, defined as the divergence of the gradient of the function u, capturing spatial changes in systems such as fluid dynamics [29].

#### Stochastic Differential Equations (SDEs).

SDEs extend ODEs by modeling the evolution of a state variable x(t) through the incorporation of randomness, often via a Wiener process $W_{t}$ [30]:

(2) $$dx\left(t\right)=\mu \left(x\left(t\right),t\right)dt+\sigma \left(x\left(t\right),t\right)dW_{t},$$

where μ and σ are drift and diffusion terms. SDEs model systems with inherent randomness such as biological processes [31].

#### Neural Differential Equations.

Neural Differential Equations extend classical differential equations by parameterizing the evolution function with neural networks. A prominent example is Neural Ordinary Differential Equations [14], where a neural network models the derivative of a latent state:$\frac{dx}{dt}=f_{\theta }(x,t)$, where $f_{\theta }$ is a neural network parameterized by θ. An ODE solver is used to compute the solution at any desired time point: $x(t)=x\left(t_{0}\right)+\int_{t_{0}}^{t}f_{\theta }(x(\tau ),\tau )d\tau$, where $t_{0}$ denotes the starting time point. For back-propagation, NODEs use the adjoint sensitivity method [32] to solve a second ODE backward in time to compute gradients efficiently: $\frac{da}{dt}=-a^{T}\frac{\partial f_{\theta }}{\partial x}$, where $a=\frac{\partial L}{\partial x}$ is the adjoint state [14]. This approach enables training with constant memory cost.

### 2.3 Combining GNNs with DEs

**Figure 1:**
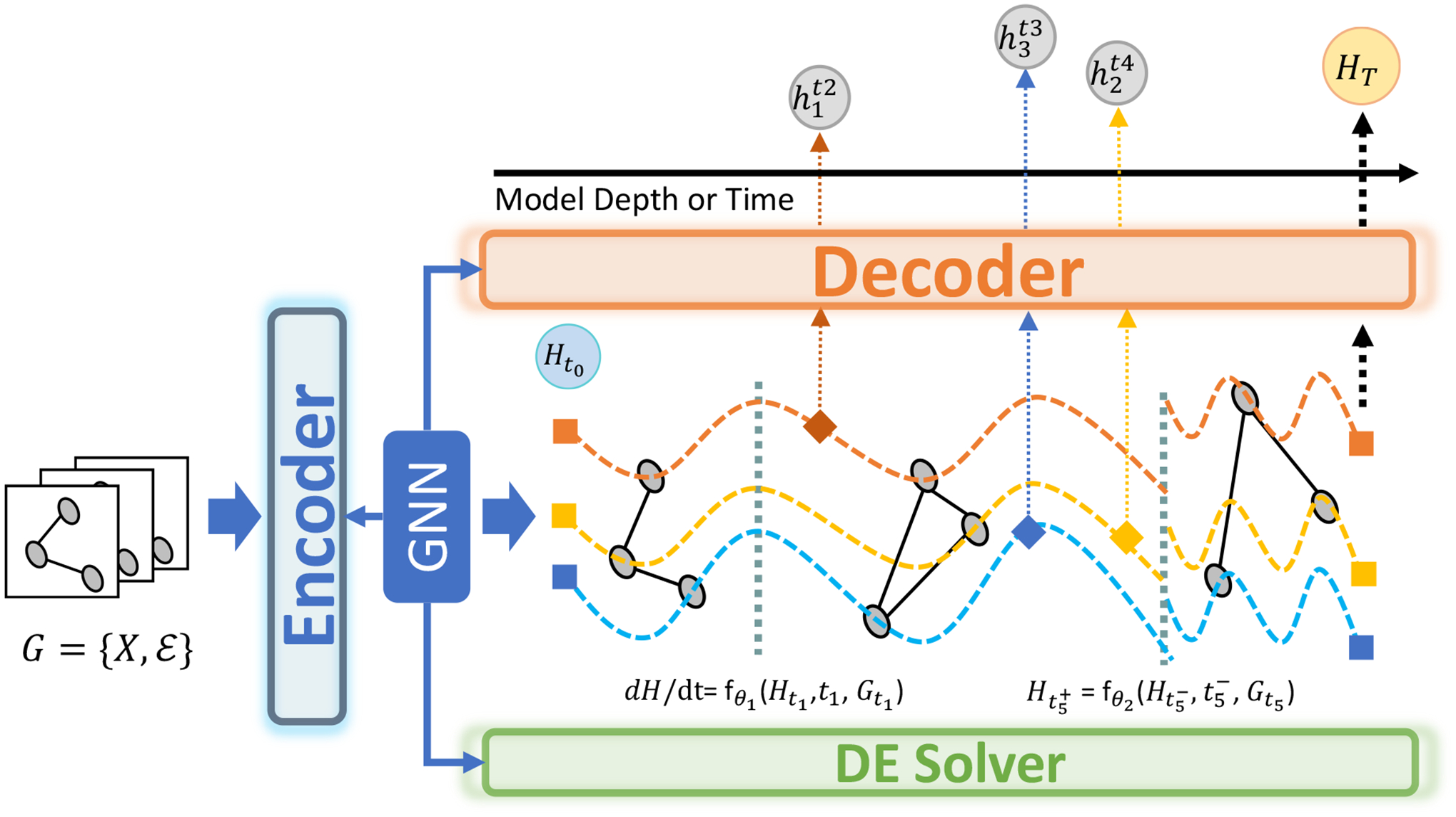
GNNs can function as an *encoder*, *decoder*, or *differential equation* in the Graph NDEs. Firstly, the encoder maps inputs to a latent initial condition, which is then propagated by the DE solver over time or model depth. Furthermore, intermediate updates can modulate state evolution through state derivatives or direct reallocation. Finally, the decoder reconstructs the latent trajectory into the target space.

By integrating the representational power of GNNs with the dynamic modeling capabilities of DEs, we introduce the concept of Graph Neural Differential Equations (Graph NDEs). A Graph NDE typically consists of two fundamental components: (i) a system of differential equations governing the temporal and spatial evolutions of states, parameterized by Neural Networks, and (ii) an initial condition that specifies the starting state of the system. While DEs establish the continuous or discrete dynamical progression of states, the role of GNNs is more flexible, as they can be incorporated at different stages of the modeling framework. As depicted in Figure 1, Graph NDEs can be roughly categorized based on the manner in which GNNs are embedded into the system dynamics.

#### 2.3.1 Roles of GNNs.

Neural DEs generally operate in a latent space, where they model the evolution of states over time. Consequently, an encoding-decoding mechanism is typically employed: an encoder maps raw input data to a latent representation, and a decoder maps the evolved latent states into the target space. GNNs can be incorporated at various points in this pipeline, functioning as encoders and decoders and parameterizing the governing DEs.

##### GNNs as Encoders.

When GNNs function as encoders, they map node feature $X\in R^{N\times D}$ into latent representations $H\in R^{N\times D^{\prime }}$ while preserving relational dependencies in the graph 𝒢 [33–36]. The encoding function can be expressed as: $H=GNN\left(X,𝒢;\Theta_{enc}\right)$, where $\Theta_{\text{enc}}$ denotes the parameters of the GNN encoder. Related works usually incorporate spatial-temporal GNNs [37, 38], capturing both the structural and temporal information.

##### GNNs as Decoders.

Given a latent representations H(t) evolved over time using DEs, a decoder maps it to the target space via: $\overset{ˆ}{Y}=GNN\left(H(t),𝒢;\Theta_{dec}\right)$, where $\Theta_{dec}$ is the decoder parameter. Graph-based decoders enable mapping that preserves node interactions and adapts to changes to graph topology [39–42].

##### GNNs as Differential Equations.

Beyond serving as encoders or decoders, GNNs can be directly embedded within the differential equation to govern continuous state flows [19, 43–46]. Let x(t) denote the state of nodes at time t. The dynamics of x(t) can be described by a GNN-parameterized differential equation, taking Graph Neural ODE as an example: $\frac{dx(t)}{dt}=GNN(x(t),𝒢,\Theta )$, where Θ is the parameter of the GNN that models continuous aggregation across the graph. Similarly, higher-order formulations extend this principle as: $\frac{d^{k}x(t)}{dt^{k}}=GNN(x(t),𝒢,\Theta )$, where k denotes the order of the DE. Such formulation enables enhanced expressiveness and adaptability in evolving graph structures [12, 47–54].

#### 2.3.2 Initial Condition Construction.

The definition of the initial condition $x\left(t_{0}\right)$ significantly influences the trajectory of the learned dynamics. Besides latent encodings, initial states can be derived from raw inputs or learned embeddings.

##### Encoding-Based Initialization.

In this case, raw node features X are first mapped into a latent space before being used as the latent initial condition, which can be deterministic: $H\left(t_{0}\right)=f\left(X,𝒢;\Theta_{\text{enc}}\right)$ or sampled from a given distribution [37, 38, 44, 55] (e.g. Gaussian): $H\left(t_{0}\right)\sim 𝒩(\overset{ˆ}{\mu },\overset{ˆ}{\sigma })$, where $\overset{ˆ}{\mu }$ and $\overset{ˆ}{\sigma }$ are inferred by a function f(X,𝒢), introducing stochasticity into the initial states to model uncertainty.

##### Pre-defined Initialization.

In physical systems, the initial condition is often dictated by domain constraints, leading to $H\left(t_{0}\right)=X$, where no additional encoding is applied. This approach is common in dynamic simulations [56–58], where initial states are predefined or randomly generated.

##### Learning-Based Initialization.

Instead of explicitly defining initial states and encoding raw features, it is also viable to learn the initial condition during model training. The model learns an optimal embedding $H\left(t_{0}\right)$ that best facilitates downstream tasks: $H\left(t_{0}\right)={\text{argmin}}_{H\left(t_{0}\right)}ℒ\left(ℱ\left(H\left(t_{0}\right),𝒢\right)\right)$, where ℒ represents a task-specific objective function and ℱ denotes the differential equation dynamics. This strategy is particularly effective in recommendation systems [59–61], where graph structure alone is available, and node representations must be inferred from relational interactions.

## 3 Taxonomies

**Figure 2:**
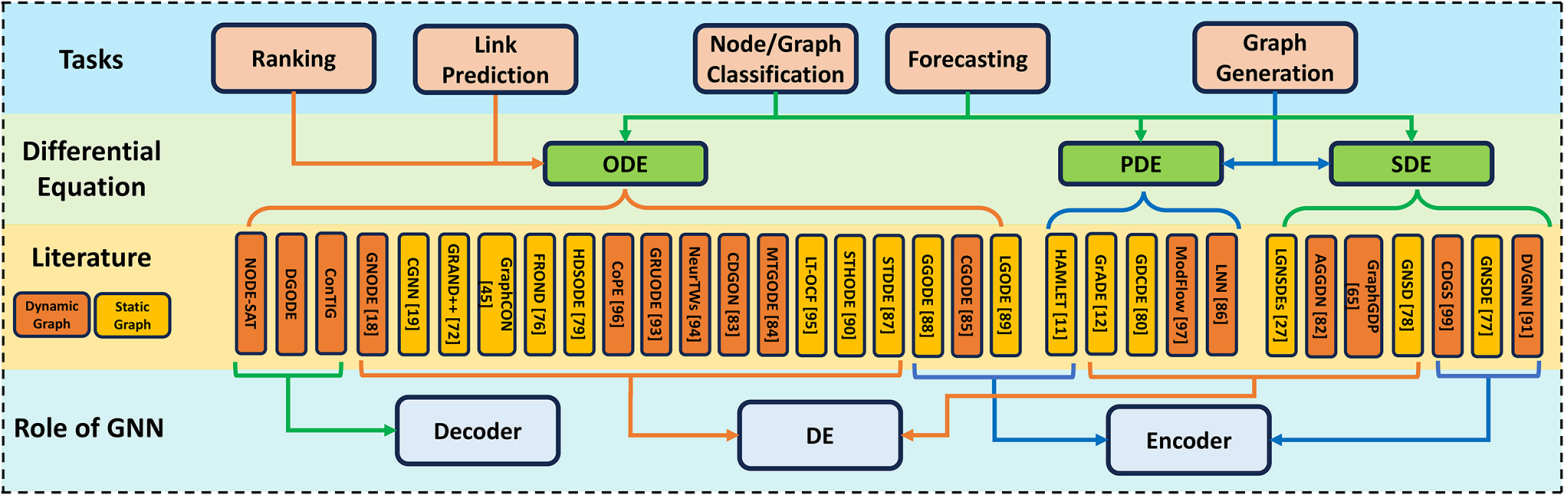
Summary of Graph Neural Differential Equation methods.

We classify Graph NDEs based on tasks, datasets, graph construction techniques, and methodological distinctions. A complete categorization is on our GitHub page, with a partial version in Figure 2.

### 3.1 Tasks

Among all the research investigated in this paper, Graph NDEs are applied in five primary tasks: *Node/Graph Classification, Link Prediction, Ranking, Forecasting*, and *Graph Generation*.

#### Node/Graph Classification.

Given 𝒢=(𝒱,ℰ), the goal is to learn functions f:𝒱→𝒴 (node classification) or g:𝒢→𝒴 (graph classification). Graph NDEs model message passing as a continuous process rather than a one-step discrete propagation. For citation network prediction [62], articles (nodes) and citations (edges) form citation graphs, where Graph NDEs improve representation learning by capturing continuous citing patterns.

#### Link Prediction.

Given 𝒢=(𝒱,ℰ), link prediction learns h:𝒱×𝒱→[0,1] to estimate edge existence probability. Graph NDEs enhance prediction by modeling continuous node embedding dynamics. Such a task is commonly seen in recommendation systems and knowledge graphs [54, 63].

#### Ranking.

Ranking assigns scores to nodes, optimizing s:𝒱→R for ordered retrieval. Graph NDEs leverage continuous diffusion to model information propagation. For example, in recommendation systems [35], users and items form bipartite graphs; Graph NDEs capture evolving preferences, refining interaction prediction.

#### Forecasting.

For dynamic graphs 𝒢(t)=(𝒱,ℰ(t)) with temporal node features X(t), forecasting estimates future states via $f:𝒱\times R^{s}\to R^{z}$, where s denotes the history input size and z refers to the horizon of prediction. Graph NDEs, incorporating time-continuous dynamics, excel in capturing gradual state transitions. For example, in traffic flow forecasting [46, 64–66], road networks use Graph NDEs for real-time flow updates and long-term forecasting.

#### Graph Generation.

Given a training set ${𝒢}_{i}$, graph generation models p(𝒢) to sample structurally meaningful graphs. Graph NDEs enable continuous latent space exploration, improving structural coherence and diversity in generated samples [67, 68].

### 3.2 Graph Construction

The construction of graphs plays a crucial role in shaping both the design and performance of Graph NDEs. This process can be analyzed along two key dimensions: *spatial* and *temporal*.

#### 3.2.1 Spatial Level.

In this paper, we depict *spatial-level relations* as a general notion of proximity or relationships between nodes. Depending on how nodes and edges are formulated, graph construction typically follows one of two primary approaches:

##### Point-Based Graphs.

Point-based graphs can be irregular, where nodes correspond to individual data points, and edges are established based on a function $f_{e}:𝒱\times 𝒱\to \{0,1\}$ that determines connectivity based on proximity from observations. The notion of proximity can be defined in various ways depending on the nature of the data, often reflecting spatial [69], or semantic relationships [70] among nodes. To quantify the strength of connectivity, edge weights can also be applied.

##### Grid-Based Graphs.

A grid-based graph is a spatially regular, where $𝒱\subset Z^{n}$ represents nodes positioned at integer lattice points in an n-dimensional space, and edges ℰ connect nodes based on a predefined neighborhood structure. This structured representation is widely used in drone swarming [71], and physical modeling [11–13, 47], where data is arranged in a spatially regular manner.

#### 3.2.2 Temporal Level.

Temporal graphs evolve over time in terms of node features or graph topology. Therefore, each time point yields a distinct graph 𝒢(t)=(𝒱(t),ℰ(t)). Formally, temporal graphs can be categorized into:

##### Static Graph.

The temporal evolution of a static graph 𝒢=(𝒱,ℰ) is solely captured through time-dependent node attributes X(t). That is, each node v∈𝒱 has a feature vector $x_{v}(t)$ evolving over time, while the edge set remains unchanged, i.e., ℰ(t)=ℰ,∀t. Applications span citation [19] and traffic [66] networks.

##### Dynamic Graph.

A dynamic graph is characterized by time-evolving edges and edge weights, ℰ(t), meaning both connectivity and interaction strengths change over time. The structure of such graphs is determined via: i) explicit modification of adjacency relations, where $ℰ(t)=\left\{e_{uv}(t)\right\}$ updates based on new inputs; ii) adaptive learning of edges and weights during training, where adjacency matrices are replaced by a learned attention matrix $A_{t}=\left(a_{ij}\right)\in R^{N\times N}$, with $a_{ij}=f\left(v_{i},v_{j},t\right)$ capturing dynamic influence. The temporal evolution of node states follows Graph NDEs: $\frac{dx(t)}{dt}=f_{\theta }(x(t),ℰ(t))$, which model node state evolution under varying graph structure. Applications span social interactions [33], graph generation [67, 68], etc.

### 3.3 Modeling Spatial & Temporal Dynamics

Graph NDEs surpass discrete models by providing a continuous flow of latent states across spatial and temporal dimensions, and the key lies in the modeling of *spatial* and *temporal dynamics*.

#### Temporal Dynamics Modeling.

In classical NDEs, the continuous evolution of variable states is defined with respect to actual time, $t\in R^{+}$, ensuring alignment with the target trajectory [14]. Specifically, in Graph NDEs, the state of each node, x(t), evolves according to a time-dependent differential equation $\frac{\text{d}x(t)}{\text{d}t}=f_{\theta }(x(t),t)$, where the graph structure is either encoded in the initial condition $x\left(t_{0}\right)$ [34] or integrated into the function $f_{\theta }$. Additionally, temporal dynamics are influenced by external controls or new inputs, which can update node states and introduce new graph structures, thereby altering the flow of states over time. Spatial-temporal models extend NDEs by explicitly incorporating the spatial dimension, capturing both spatial and temporal evolution on dynamic graphs.

#### Spatial Dynamics Modeling.

The dynamic spatial evolution can also be applied to static graphs, where the model *depth* [20] corresponds to a continuous notion of time. Unlike the conventional approach of stacking discrete GNN layers, this continuous perspective naturally connects to diffusion equations [72]. A general graph diffusion equation is given by:

(3) $$\frac{\partial x(t)}{\partial t}=\text{div}(G(x(t),t)\cdot \nabla x(t)),$$

where ∇x(t) is the divergence of x,G(x(t),t) is the diffusion coefficient that may depend on both the current state x(t) as well as current time or depth t, and div(⋅) denotes the graph divergence operator. By parameterizing this diffusion process, we arrive at the formulation of NDEs on graphs.

Recent work provides further insight into this connection. For instance, Chamberlain et al. [73] show that GNNs can be viewed as the discrete form of Beltrami flow, while Choi et al. [74] develop a reaction-diffusion-based GNN architecture. Taken together, these studies reinforce the link between Graph NDEs and graph diffusion processes. Consequently, Graph NDEs provide both smoother feature propagation across the graph and a principled physical analogy grounded in a well-established diffusion process.

## 4 Methodology

In this section, we detail the methodology underpinning Graph Neural Differential Equations (Graph NDEs). Our discussion is organized around two primary perspectives: *Temporal Dynamics Modeling* and *Spatial Dynamics Modeling*. For each perspective, we elaborate the unique challenges involved.

### 4.1 Temporal Dynamics Modeling

For spatial-temporal models, incorporating the time dimension presents several challenges. This section outlines key temporal modeling challenges and corresponding solutions in Graph NDEs, including *dynamic updates, irregular time intervals, modeling temporal delay, modeling hybrid system dynamics*, and the *efficiency*.

#### 4.1.1 Dynamic Temporal Updates.

For a naive DE, whether parameterized by an NN or not, a fixed trajectory is predicted once the initial condition is given. This is because the DE defines the evolution or flow of node states, namely the vector field [14]. However, in a spatial-temporal graph, both node features and the graph structure can evolve over time independently of the vector field defined by the DE. These updates significantly impact the target trajectory. From the perspective of the vector field, the updated graphs can be interpreted in two ways: 1. Reallocation of states, and 2. Conditioned states flow. For the reallocation of states, the vector field does not change while the new inputs introduce a jump of states in the vector field: $x\left(t_{k}^{+}\right)=\Phi \left(x\left(t_{k}^{-}\right),\Delta \left(t_{k}\right)\right)$, where $t_{k}$ denotes the moment of a discrete update, $t_{k}^{-}$ and $t_{k}^{+}$ are the times just before and just after the jump, respectively, and $\Delta \left(t_{k}\right)$ captures the new information or structural change at $t_{k}$. The update function Φ then adjusts the node states accordingly. This jump effectively resets the trajectory of the node states, altering the dynamics governed by the DE. For example, Poli et al. [19] introduced an autoregressive graph differential equation that applies “jumps” to adapt to the dynamic graph structure., and Zhang et al. [75] applied Graph NDE in the recommendation system, where jumps of node features are introduced as the interaction between the user and item changes.

In the case of a conditioned state flow, newly arrived inputs at a given time point serve as the input of Graph NDEs, which gives a formulation of $\frac{dX}{dt}=F(X(t),𝒢(t))$, where X(t) is the current node states and 𝒢(t) is the dynamic graph including node features and the graph structure. The change of 𝒢(t) can be viewed as either a change of the vector field or as an adjustment to the output $\frac{dX}{dt}$. Instead of solely relying on the initial condition, the vector field becomes conditioned on the current input, allowing the system to adapt its trajectory in response to external influences [76].

#### 4.1.2 Irregular Time Interval.

Real-world dynamic systems exhibit irregularly sampled time series, where observations occur at non-uniform intervals, making traditional discrete-time models ineffective. DE-based models naturally address this by modeling the continuous evolution of node states so that the states at arbitrary time points can be inferred. Recent advancements, such as LG-ODE [77], CG-ODE [78], and GG-ODE [79], extend such ability to graph-structured data. LG-ODE [77] models continuous node dynamics using latent ODEs, enabling interpolation across uneven time steps. CG-ODE [78] further generalizes this by incorporating evolving graph structures, where both node states and edge interactions are learned through coupled differential equations. GG-ODE [79] extends these ideas across multiple environments by introducing environment-specific latent factors, enabling the transfer of learned dynamics across different systems. These approaches provide a flexible framework for modeling real-world graph dynamics under irregular sampling, outperforming discrete-time methods in handling asynchronous, partially observed data.

#### 4.1.3 Temporal Delay Modeling.

Traditional GNNs assume immediate information propagation, which fails to capture the inherent temporal delays present in real-world systems. In applications such as traffic forecasting, changes in one location take time to influence others, making delay-aware modeling essential. Long et al. [80] introduces the Spatial-Temporal Delay Differential Equation, which explicitly incorporates time delays into spatial-temporal modeling. The core idea is to model node interactions as: $\frac{dh_{i}(t)}{dt}=f\left(h_{i}(t),h_{j}\left(t-\tau_{ij}\right),\theta \right)$, where $\tau_{ij}$ represents the time delay in information propagation between nodes. Instead of assuming fixed delays, they propose two approaches: (1) a precomputed delay estimator using *max-cross correlation*, and (2) a time-delay estimator that dynamically adjusts $\tau_{ij}$ based on traffic conditions.

#### 4.1.4 Temporal Dynamics for Hybrid Systems.

While Graph NDEs parameterize the DEs with NNs or GNNs, making the model inherently data-driven, incorporating domain-specific biases can be beneficial for regularizing the model outputs. Such modeling biases, often derived from well-established physical laws, constraints, or expert knowledge, are fundamental in traditional knowledge-driven models. To enhance the effectiveness and interpretability of Graph NDEs, hybrid models [6] integrate domain-knowledge into the modeling of Graph NDEs by either *constraining the form of Graph NDEs* or *predicting the abstract quantities or parameters of the physical system*. Li et al. [81] extend the use of the spatial-temporal decay model from one-dimensional dynamics to the high-dimensional latent space. Similarly, Han et al. [82] make use of the structure of the Susceptied-Infected-Recovered model and switch the modeling to the latent space. On the other hand, Sanchez-Gonzalez et al. [83] model the Hamiltonian mechanism by predicting the momentum and velocity in the physical function using a GNN. Similar practices have been made for Lagrangian mechanism [84].

#### 4.1.5 Efficient Temporal Simulation.

Although classic PDE models effectively describe various real-world phenomena with numerical solvers such as the Finite Element Method [85], time complexity remains a significant challenge, particularly for solving complex dynamics and real-time processing tasks [86]. The primary sources of this challenge are twofold. First, the high dimensionality and non-linearity of many problems lead to more intricate PDE systems, both in terms of the number of equations and their structural complexity. Second, the demand for larger or more fine-grained grid-based graphs further increases the number of nodes to be modeled [87], which can be computationally expensive using classic PDE solvers. Beyond the computational inefficiency of handling large-scale and complex PDEs, additional challenges arise when dealing with problems that lack a fixed PDE formulation. To address these issues, Graph Neural PDEs [11–13, 47] employ a data-driven approach to learn and integrate the governing rules of both spatial and temporal dynamics across all nodes using a single model. Unlike traditional methods, where computational complexity scales with problem intricacy and the graph size, Graph Neural PDEs maintain a fixed model for all cases and does not rely on classic PDE solvers. As a result, the time complexity does not increase with the problem complexity, and the governing dynamics are encapsulated within the learned parameters, enabling efficient and scalable solutions.

### 4.2 Spatial Dynamics Modeling

Unlike spatial-temporal dynamics which model latent state evolution over time, spatial dynamics can also be modeled in terms of model depth. While traditional GNNs effectively capture static relationships through local aggregation, their discrete nature constrains their ability to represent continuous feature evolution. Here, we show how embedding GNNs in differential equations enables dynamic graph modeling and overcomes classic challenges such as *over-smoothing, measuring uncertainty, adversarial robustness, graph heterophily*, and *modeling high-order relations*.

#### 4.2.1 Over-Smoothing on Graphs.

GNNs with deeper architecture experience severe performance degradation due to vanishing gradient and over-smoothing, where node representations become indistinguishable and converge to the same value as more layers are added [88]. To mitigate the effect of over-smoothing, previous studies imitate residual networks [89] and develop skip connections [90] between layers, updating the node features using $H(t+1)=H(t)+{\text{GNN}}_{\theta }(H(t),𝒢)$, where t denotes the layer index. On the other hand, Graph NODE [19] takes a step further by making this process differential: $\frac{dH(t)}{dt}=\text{GNN}(H(t),𝒢)$. Then, a numerical solver [91] is applied to acquire the trajectory. Additionally, CGNN [20] introduces the initial latent embeddings, $E=H\left(t_{0}\right)$, into the ODE formulation: $\frac{dH(t)}{dt}=\text{GNN}(H(t),𝒢)+H\left(t_{0}\right)$. This results in a function with a restart distribution, which helps the model retain the initial representations and effectively mitigates over-smoothing. Moreover, GRAND++ [92] interprets over-smoothing through the lens of diffusion, where deeper networks excessively diffuse node features, ultimately leading to uniform feature representations across all nodes. Similar to CGNN, Grand++ mitigates this issue by introducing a source term (restart) in the differential equation (DE) to preserve initial representations. Likewise, Graph-Coupled Oscillator Networks [48] establish a connection between over-smoothing and zero-Dirichlet energy steady states, proposing a second-order ODE to counteract the over-smoothing. Furthermore, Maskey et al. [93] extend the problem to directed graphs and tackle over-smoothing with fractional graph Laplacians.

#### 4.2.2 Uncertainty within Graph Dynamics.

Real-world data often contains noise and unobserved external factors that influence the dynamics of graph propagation. For one thing, graphs are often constructed from real-world data where the connections (edges) between nodes can be incomplete, noisy, or even spurious [94]. For another thing, even though a clear graph structure is given, the way information, influence, or any form of signal diffuses through these networks can be highly variable and subject to external factors (e.g., weather, human behavior) [95]. Since GNNs and Graph NODEs make predictions conditioned on neighbors, both kinds of uncertainty impact their performance. To provide a direct measurement of uncertainty and improve the robustness of these models, Graph Neural SDEs introduce a stochastic diffusion term $\sigma (x(t),t)dW_{t}$, as illustrated in Equation 2, enhancing the performance on node classification tasks in both the In Distribution and Out of Distribution cases [30, 96, 97]. Furthermore, Liang et al. [98] combines graph variational encoding with SDE, generating dynamic graphs for spatial-temporal forecasting. Xing et al. [76] stacked the SDE module upon ODE, which works as a control signal to modulate the SDE propagation. Huang et al. [68] applied SDE in the graph generation task by applying the reverse-time SDE to generate the target permutation-invariant graphs from random graphs.

#### 4.2.3 Graph Adversarial Robustness.

GNNs are vulnerable to adversarial perturbations due to inter-node information exchange. Adversaries can perform modification attacks by adding or removing edges or injection attacks by introducing malicious nodes. Song et al. [99] treat graphs as discretized Riemannian manifolds and analyze the stability of the heat kernel under metric perturbations. Their results show that for small perturbations ε=o(1), the change in node features remains bounded $\left\|\varphi (u,t)-\tilde{\varphi }(u,t)\|=O(\varepsilon \right)$, where φ(u,t) is the node attribute of node u at time t, indicating that PDE-based GNNs can better withstand adversarial topology attacks. Building on this analysis, they propose a novel class of graph neural PDEs with stronger defenses against such adversarial modifications. While Song et al. [99] demonstrate Lyapunov stability, it does not necessarily guarantee adversarial robustness. Zhao et al. [100] analyze various stability concepts for graph neural flows, leading to the Hamiltonian Graph diffusion class, which improves robustness by maintaining constant total Hamiltonian energy over time, ensuring bounded BIBO stability. Recently, Kang et al. [101] made an extension to graph neural fractional-order differential equations, showing more robust than existing Graph neural ODEs.

#### 4.2.4 Graph Heterophily.

GNNs have been widely used for various graph-based learning tasks, yet they often assume connected nodes have similar attributes (homophily), which is not hold in heterophilic graphs, leading to suboptimal performance. To address this, recent works have explored Neural ODEs combined with graph dynamic modeling to enhance node representation learning in heterophilic settings. Recent studies have introduced diffusion-based models to handle heterophilic graphs effectively. Zhao et al. [102] propose a Graph Neural Convection-Diffusion framework, leveraging the convection-diffusion equation (CDE) to incorporate both homophilic and heterophilic information. The convection-diffusion equation is formulated as: $\frac{\partial x}{\partial t}=\text{div}(D\nabla x)-\text{div}(vx)$, where the first term represents diffusion, and the second term accounts for convection with velocity field v controlling the propagation direction. In the discrete graph setting, this extends to: $\frac{\partial x(t)}{\partial t}=\text{div}(D(x(t),t)⊙\nabla x(t))+\text{div}(v(t)\circ x(t))$, where v(t) now adapts to node dissimilarity, enhancing classification performance on heterophilic graphs. Similarly, Zhang and Li [103] introduce a dual-channel Continuous Graph Neural Network with latent states applied using low-pass $\left(H_{L}\right)$ and high-pass ${(H}_{H})$ filtering :

(4) $$\frac{\partial H_{L}}{\partial t}=\left({\overset{ˆ}{A}}_{\text{sym}}-I\right)H_{L}+H\left(t_{0}\right),\;\frac{\partial H_{H}}{\partial t}=\left(-{\overset{ˆ}{A}}_{\text{sym}}\right)H_{H}+H\left(t_{0}\right).$$

where ${\overset{ˆ}{A}}_{\text{sym}}$ is the symmetrically normalized adjacency matrix, and the features are mixed from both channels in the end.

#### 4.2.5 Graph Dynamics with High-Order Relations.

Many real-world problems involve interactions that go beyond pairwise relationships. Hypergraph learning [104] addresses this by allowing each hyperedge to connect multiple nodes. Nevertheless, incorporating hypergraph learning into the framework of Graph NDEs is non-trivial as the dynamics of pair-wise graphs and hypergraphs are different. To bridge the gap, Yao et al. [105] propose to model spatial and temporal evolutions separately, building a spatial hypergraph $G_{sp}$ and a temporal hypergraph $G_{te}$. Then, hypergraph convolution is integrated into the ODE, which yields the spatial and temporal evolutions on the two graphs. In the end, an MLP layer is applied to combine the final embeddings from the spatial and temporal levels. Besides separately encoding the spatial and temporal evolutions, Yan et al. [106] propose to encode the node embeddings $H_{v}$ and hyperedge embeddings $H_{e}$ separately, which gives an ODE in the form of: $\left[\begin{array}{c} {\dot{H}}_{v}\\ {\dot{H}}_{e} \end{array}\right]=\left[\begin{array}{l} g_{v}\left(H_{v}(t)\right)\\ g_{e}\left(H_{e}(t)\right) \end{array}\right]+A\left[\begin{array}{l} H_{v}(t)\\ H_{e}(t) \end{array}\right]$, where $g_{v}$ and $g_{e}$ are the control functions and A denotes the diffusion velocity effect between the vertex representation and the hyperedge representation in the dynamic system by the correlation of the hypergraph.

## 5 Applications

Graph NDEs have been applied across various domains due to their capability to model continuous spatial and temporal dynamics. In this section, we discuss on some popular applications, including *Physics Systems Simulation*, *Traffic Flow Forecasting*, *Recommendation Systems*, *Epidemic Modeling*, and *Graph Generation*.

### 5.1 Physics Systems Simulation

Graph Neural ODEs have proven effective for modeling continuous-time dynamics in physics-based simulations by parameterizing system evolution through differential equations, allowing flexible and efficient trajectory prediction. These models are particularly useful for simulating multi-body interactions [79], particle dynamics [107], spring system [44], charged particle system [44], chaotic pendulum system [108] and fluid mechanics [109], where relational structures naturally fit graph representations. For example, the Hamiltonian Graph Network [110] incorporates Hamiltonian mechanics to enforce energy conservation in learned physics models. GG-ODE [79] introduces environment-specific latent factors to adapt physics models across different conditions. EGODE [58] extends Graph Neural ODEs to hybrid systems, handling sudden state changes, e.g.,rigid-body collisions. Furthermore, GNSTODE [111] improves spatial-temporal modeling in physics systems by learning latent force interactions and refining long-range dependencies. These models demonstrate how Graph ODEs enhance the accuracy, efficiency, and generalization of physics-based simulations, outperforming numerical solvers in long-term stability and adaptability.

### 5.2 Traffic Flow Forecasting

Traffic flow forecasting is a crucial task in intelligent transportation systems, requiring models that can capture complex spatial-temporal dependencies. Traditional models, such as ARIMA and LSTM-based approaches, struggle with irregular traffic patterns and evolving road network dynamics. Graph NDEs provide a continuous-time framework that integrates spatial-temporal dynamics, improving long-range forecasting and adapting to variable time intervals. For example, STGODE [112] models traffic flow as a continuous dynamical system, integrating GNNs with an ODE solver to handle long-term dependencies. GODE-RNN [113] combines Graph NDEs with RNNs, capturing both fine-grained temporal changes and spatial interactions. ASTGODE [114] introduces an attention mechanism within Graph ODEs to enhance interpretability and adaptive forecasting. Additionally, GRAM-ODE [115] employs multiple Graph ODE modules to learn hierarchical traffic patterns, while AGODE [116] dynamically updates the graph structure to reflect changing traffic conditions. These methods outperform discrete-time GNN models in accuracy, demonstrating the potential of Graph NDEs to handle irregular and evolving traffic data efficiently.

### 5.3 Recommendation Systems

Recommendation systems [117] naturally form a bipartite graph structure, capturing relationships between users and items. Graph NDEs effectively model the continuous evolution of user preferences, surpassing traditional collaborative filtering methods [118] by accounting for dynamic interactions. For example, Qin et al. [119] propose an autoregressive propagation framework with an edge-evolving mechanism and a temporal aggregation module to predict user-item interactions, which is similar to CoPE [75] and Con-TIG [42]. To further enhance the learned representations of Graph NDEs, Yang et al. [120] integrate contrastive learning into their optimization process. Additionally, to improve adaptability to dynamic graphs, Guo et al. [35] propose t-Alignment, which synchronizes the updating time steps of temporal session graphs within a batch.

### 5.4 Epidemic Modeling

Modeling infectious disease spread [6] is crucial for public health policy design. In an epidemic graph, nodes represent individuals or communities, while edges denote their interactions. Graph NDEs extend beyond the traditional mechanistic models, e.g., SIR and its variants, introducing *hybrid models* [6] that integrate both GNNs and mechanistic models. These approaches enhance the ability to capture complex infection dynamics. For example, STAN [121] preserves the ODE function of the SIR model and uses GNNs to predict the parameters of the SIR model on a static graph. As an extension, MepoGNN [122] adopts a graph learning module, which introduces learning on dynamic graphs. Besides learning the parameters of mechanistic models, Wan et al. [123] integrate the ODE function of the SIR model with GNNs, modeling the variables in the high-dimensional latent space.

### 5.5 Graph Generation

Graph generation is essential for applications like drug discovery and program synthesis [124], but modeling graph distributions is challenging due to their discrete, permutation-invariant nature. Traditional models like variational autoencoders [125] struggle with this invariance. Score-based generative models address this by using graph SDEs to simulate graph trajectories, where diffusion corrupts graphs into a prior distribution (e.g., normal distribution), and the trajectory captures both diffusion and denoising. These models rely on log-density gradient vector fields, imposing fewer constraints than likelihood-based approaches while ensuring permutation invariance. The edge-wise dense prediction GNN [126] estimates scores for graph distributions while maintaining permutation invariance but is limited to adjacency matrices. To overcome this, Jo et al. [127] proposed Graph Diffusion via SDEs, which models both node features and adjacency matrices with separate drift and diffusion terms, capturing node-edge dependencies. CDGS [128] further improves this with hybrid message-passing blocks and fast ODE solvers, enabling rapid, high-quality molecule generation.

## 6 Future Work

While significant advancements in Graph NDEs, many challenges remain largely unexplored. In this section, we discuss these issues and suggest directions for future research.

### 6.1 Discovering Graph Differential Equations

Equation discovery is an essential task across scientific disciplines, facilitating the extraction of explicit mathematical relationships directly from observed data [129]. At the heart of this process lies *symbolic regression*. Recently, deep learning-based methods, such as set-to-sequence transformers [130] and large language models [131], have emerged as viable alternatives to traditional symbolic regression approaches. However, due to inherent architectural constraints, they focus on discrete representations of data. In contrast, Graph NDEs explicitly capture continuous dynamical systems by learning vector field representations, yet they typically encode equations in implicit forms. This fundamental difference indicates that integrating discrete-focused transformer-based methods with continuous-based Graph NDEs presents a compelling pathway toward advancing the field of differential equation discovery.

### 6.2 Handling Graph Sparsity and Sporadicity

Data sparsity in dynamic systems remains a critical challenge, often manifesting as limited labeled nodes and missing or incomplete observations over time and space. Additionally, sporadic patterns, characterized by both sparsity and irregular, unpredictable distribution, further challenge learning and inference [132]. Recently, Luo et al. [133] combined the strengths of neural processes and neural ODEs to model evolving graphs with missing edges and to capture physical dynamics from highly sparse spatial-temporal data. Nevertheless, open challenges persist in ensuring the robustness and efficiency of Graph NDEs under extreme sparsity conditions. To further address the challenge, possible solutions may involve zero-shot annotator to label a small portion of nodes [134], or graph condensation [135] that yields a condensed graph from sparse graph.

### 6.3 Scalability on Large Dynamic Graphs

Scalability remains a significant challenge for Graph NDEs, particularly when applied to large-scale dynamic graphs. These models require the solving of continuous-time differential equations for potentially millions of nodes and edges, leading to high computational overhead and memory demands [136]. The iterative nature of DE solvers exacerbates the issue since repeated evaluations of neural network functions over numerous time steps can be prohibitively time-consuming. To tackle this challenge, several approaches can be explored: developing efficient numerical solvers tailored for neural differential equations [137], leveraging parallelization and GPU acceleration, and employing sparse representations and approximation methods [138].

### 6.4 Modeling Continuous Structural Evolution

Real-world graphs, such as social networks, frequently undergo dynamic changes, with nodes and edges continuously evolving over time. While several studies have introduced flexible approaches to incorporate dynamic inputs during inference, either by adjusting the flow direction conditioned on new inputs or jumping in the vector field (see Section 4.1.1), the evolving dynamics of graph structures have received limited attention. Most existing methods either generate graphs using SDEs in a discrete manner [98] or adopt an end-to-end approach for graph generation tasks [128]. Although Huang et al. propose a framework that allows the dynamic evolution of both edge weights and node features, it overlooks the newly observed graph structures that emerge dynamically. Since graph topology significantly influences the evolution of node embeddings, it is crucial to incorporate the dynamic evolution of graph structures during inference to improve downstream performance.

### 6.5 Modeling Hierarchical Graph Dynamics

Hierarchical or multi-scale data is crucial for capturing complex structures and long-range dependencies across different granularities. While multi-scale GNNs have been well-studied[139], their integration with neural differential equations remains underexplored. Multi-scale features can appear temporally and spatially and be incorporated at various modeling stages, such as encoding or differential equation modeling, resulting in multiple latent trajectories. For example, Wang et al.[140] encode multi-scale temporal information before applying a graph-based ODE. However, integrating multi-scale spatial modeling within differential equations is still an open research challenge in Graph NDEs.

## 7 Conclusion

This survey presents the first comprehensive overview of Graph Neural Differential Equations (Graph NDEs), starting with core concepts from GNNs and differential equations. We propose a structured taxonomy of tasks, graph construction methods, and GNN roles. Existing literature is analyzed through two main lenses: Temporal and Spatial Dynamics Modeling, with key challenges and solutions highlighted. We also explore applications and research gaps, offering future directions. By showing how GNNs integrate with differential equation frameworks, this survey aims to drive further innovation in the field.

## Supplementary Material

1
