# Supplementary material for: Graph ODEs and Beyond: A Comprehensive Survey on Integrating Differential Equations with Graph Neural Networks
Source: KDD. Author manuscript; Available in PMC 2025 Nov 3. (PMC12363673; doi:10.1145/3711896.3736559)
Supplement: 1 [file NIHMS2093258-supplement-1.pdf]

Appendices

A Summary of Graph NDEs

Table 1: Comprehensive Summary of Graph Neural Differential Equations.

Task	Paper	Role of GNN	DE Type	Graph Construction
**Node/Graph Classification**	[21, 151]	DE	First Order ODE	Dynamic
	[22, 46, 65, 96, 97, 104, 107, 152–154]	DE	First Order ODE	Static
	[51]	DE	Second Order ODE	Static
	[105]	DE	Fractional Order ODE	Static
	[100]	Encoder	First Order SDE	Static
	[33, 101]	DE	First Order SDE	Static
	[110]	DE	First Order ODE	Static
	[76, 103, 106]	DE	First Order PDE	Static
	[155]	DE	First Order PDE & Second Order PDE	Static
**Forecasting**	[79]	DE	First Order ODE & First Order SDE	Dynamic
	[80, 85, 156–166]	DE	First Order ODE	Dynamic
	[82, 167–169]	Encoder & DE	First Order ODE	Dynamic
	[39, 170, 171]	Encoder	First Order ODE	Dynamic
	[172–174]	DE	Second Order ODE	Dynamic
	[175]	Encoder	Second Order ODE	Dynamic
	[176]	Encoder & DE	Second Order ODE	Dynamic
	[177]	DE	First-order NCDE (Neural Controlled Differential Equation)	Dynamic
	[102]	Encoder	First Order SDE	Dynamic
	[88]	DE	Second Order PDE	Dynamic
	[178]	DE	First Order ODE & First Order SDE	Static
	[13]	DE	Second Order PDE	Static
	[12]	Encoder	First Order PDE	Static
	[50]	DE	High Order PDE	Static
	[84, 179]	DE	First Order DDE	Static
	[47, 81, 83, 180–182]	Encoder & DE	First Order ODE	Static
	[37, 61, 109, 111, 183–210]	DE	First Order ODE	Static
	[211, 212]	DE	Second Order ODE	Static
	[213, 214]	Encoder	First Order ODE	Static
	[215, 216]	DE	First-order NCDE (Neural Controlled Differential Equation)	Static
	[25, 217]	DE	First Order SDE	Static
	[87]	DE	First Order PDE	Static
	[197]	DE & Decoder	First Order ODE	Static
**Link Prediction**	[218, 219]	DE	First Order ODE	Dynamic
	[43]	Decoder	First Order ODE	Dynamic
**Ranking**	[220]	DE	First Order ODE	Static
	[78]	DE	First Order ODE	Dynamic
	[221]	Decoder	First Order ODE	Dynamic
**Graph Generation**	[147]	DE	First Order PDE	Dynamic
	[71, 133, 134]	DE	First Order SDE	Dynamic
	[135]	Encoder	Third Order SDE	Dynamic
